# 

**Published:** 1872-11

**Authors:** 


### ATLANTA MEDICAL & SURGICAL JOURNAL.

EDITED BY

S.G.O. INDEXED

JOSEPH P. LOGAN, M.D.,

AND

W. F. WESTMORELAND, M.D.,

*Professor of the Principles and Practice of Surgery in Atlanta Medical College.*

VOL. X.—NOVEMBER, 1872.—No. 8.

*PAX ET SCIENTIA, SED VERITAS SINE TIMORE.*

ATLANTA, GEORGIA:
HERALD PUBLISHING COMPANY'S PRESS.
1872.

